# Influence of adhesive systems on microtensile bond strength of resin-based endodontic sealers to the root dentin

**DOI:** 10.4317/jced.51028

**Published:** 2014-07-01

**Authors:** Juan B. Rodríguez-Martínez, María P. González-Rodríguez, Santiago González-López, Carmen M. Ferrer-Luque

**Affiliations:** 1 PhD, Graduate in Dentistry. Department of Dental Pathology and Therapeutics, School of Dentistry, University of Granada. Spain; 2 DDS, PhD, Associate Professor. Department of Dental Pathology and Therapeutics, School of Dentistry, University of Granada. Spain; 3 DDS, MD, PhD, Associate Professor. Department of Dental Pathology and Therapeutics, School of Dentistry, University of Granada. Spain

## Abstract

Objective: The aim of this study was to determine the microtensile bond strength to root dentin of AH Plus™ and EndoREZ® with Clearfil Liner Bond 2V and Optibond Solo™ Plus adhesive systems. Study Design: The coronal and middle thirds of six single rooted bovine teeth was split longitudinally in a mesio-distal direction. The two halves were joined with AH Plus or EndoREZ, with and without the use of Clearfil Liner Bond 2V and Optibond Solo™ Plus adhesive systems. Build-ups were vertically sectioned into quadrangular (≈1mmx1mm) compound bars and subjected to tensile tests at a constant crosshead speed (1 mm/min) until debonding. Results: Optibond® Solo Plus™ in combination with AH Plus™ and EndoREZ® showed the highest mean microtensile bond strength values, in both coronal and middle thirds. The lowest results were seen in the groups where no dentine adhesive was applied, and in those where the self-etching adhesive Clearfil Liner Bond 2V was used. Conclusion: The microtensile bond strength to root dentin of AH Plus™ and EndoREZ may be increased with the use of a total-etch adhesive.

** Key words:**Adhesive systems, AH Plus, EndoREZ, microtensile bond strength, root dentin.

## Introduction

Adhesion of endodontic sealers to root dentin walls is an important factor in providing a complete seal along the root canal system. The bond strength of endodontic sealers varies, depending on their chemical composition (1). Although the values seen with all the groups of endodontic sealers are generally low, the ones based on resins have demonstrated greater bond strength to dentin (2). Likewise, smear layer removal is known to improve the adaptation of filling materials to the root canal, thereby increasing the bond strength of resin-based endodontic sealers to root dentin (3-6).

The use of dentine bonding agents (7-9) in root canal filling was introduced to enhance the endodontic re-sin-based sealers adhesion to the root dentin. The newer endodontic methacrylate resin-based sealers [RealSeal, Epiphany] use a separate self-etching primer before application of flowable composites to the primed dentin (10); or else consist of a single product (11) the self-adhesive methacrylate sealer, incorporating a self-etching primer and a moderately filled flowable composite [MetaSEAL; RealSeal SE, Epiphany SE]. However, the bond strength of these sealers is reportedly not superior to that found with epoxy resin endodontic sealers (12). Some studies have shown that the microleakage is reduced when total etching and self-etching adhesive systems are used (13,8) and a greater adhesive strength to root dentin of both an epoxy resin sealer [AH26] and a dimet-hacrylate sealer [EndoREZ®] (14,15).

The aim of this study was to assess the microtensile bond strength to bovine root dentine of AH Plus™ and EndoREZ® resin-based sealer cements, with two bonding strategies, self-etching and total-etch adhesive systems. The null hypotheses tested were: (1) resin-based sealer cements, AH Plus™ and EndoREZ®, have not differences in the microtensile bond strength to root bovine dentin (2) the use of a self-etching, Clearfil Liner Bond 2V or a total-etch, Optibond Solo™ Plus, adhesive systems increased the microtensile bond strength to root dentin in both sealer cements.

## Material and Methods

Six bovine incisors with no signs of structural anomalies were selected after careful visual inspection and stored in 0.2% thymol at 37ºC. Before use, they were cleaned with ultrasonic scalers and washed several times in water.

- Specimen preparation

The crowns were cut off perpendicular to the long axis at 1mm above the cementum-enamel junction and their apices were sectioned at 4mm with a low-speed diamond disk saw [Struers A/S, Ballerup, Denmark] under running water. The remaining section of the roots was split longitudinally in a mesio-distal direction with the diamond disk saw. The portions of the root surface where the canal had been located were ground flat against #600 grit silicon carbide paper discs [WS 18-B, Struers, Ballerup, Denmark].

The external surfaces of the roots were ground using a 150-grit silicon carbide paper in order to create a rough surface, then they were etched with 37% phosphoric acid gel for 30s [Ivoclar Vivadent, Schaan, Liechtenstein], conditioned with SoloBond M adhesive [VOCO GmbH, Cuxhaven, Germany] following manufacturer´s instruction and covered with 3 layers of Synergy® Duo Shade composite [Coltène/Whaledent, Cuyahoga Falls, OH, USA] until forming a rectangular block 1cm high. Each layer was polymerized for 45 seconds with a Astralis 10 lamp at 700 mW/cm2 [Ivoclar Vivadent, Schaan, Liechtenstein] and the external surface of the blocks was polished with a 220-grit silicon carbide paper discs.

- Dentin treatment and bonding procedures

Two blocks of a single root [n=6] were adhered to each other to obtain the bars specimens [n=219] as shown in  Table 1. The dentine adhesives Optibond Solo™ Plus [Kerr Corporation, Orange, CA, USA] and Clearfil Liner Bond 2V [Kuraray Europe GMBH, Frankfurt, Germany] were applied following manufacturer’s instructions. Smear layer was removed with gel phosphoric acid at 37% [Total Etch®, Ivoclar Vivadent. Schaan, Liechtenstein] for 15s washed for 10s and wet-dried with air in the no adhesive and Optibond Solo™ Plus groups. AH-Plus™ [Dentsply DeTrey, Konstanz, Germany] and EndoREZ® [Ultradent Products, South Jordan, UT, USA] were applied in each half following manufacturer´s instructions. After applying an established amount of the endodontic sealer, the two halves were put together and a pressure of one kilogram was applied in order to obtain a thin layer of the sealer cement. Then the specimens were stored at 37ºC for 72 hours in order to allow the setting of the sealers.

**Table 1:**
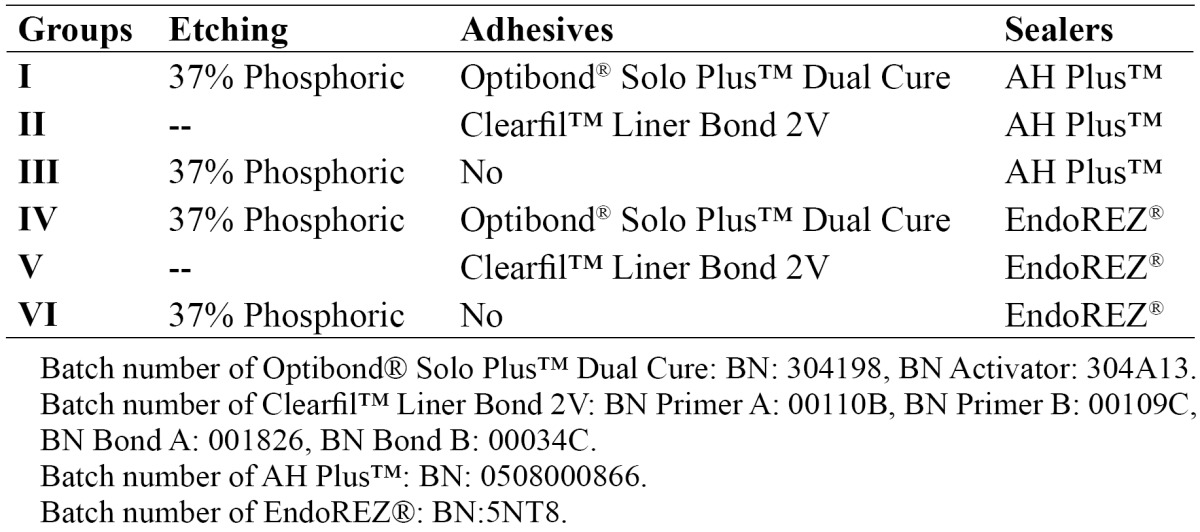
Study groups.

The build-ups were vertically sectioned into quadrangular [≈1mmx1mm] compound bars with a hard tissue microtome Accutom-50 [Struers, Ballerup, Denmark]. The tops and bottoms of the bars were made up of dentine and the middle was sealer. These bars were glued using cyanoacrylate adhesive gel [Henkel Adhesivos, Barcelona, Spain] to a probe and submitted to failure in tension using a universal testing machine [Ibertex Electrotest 500, Madrid, Spain] at constant crosshead speed [1 mm/min] until debonding. A digital calliper with an accuracy of 0.001 mm [Mitutoyo Corporation, Aurora, IL, USA] was used to measure the sides of the bonding interface and calculate the bonding area in mm2. Microtensile bond strength [µTBS] data were expressed in MPa. Pre-testing failure during section, manipulation or fixation processes were not counted. Fractured surfaces were inspected [40X magnification] in a stereo microscope [Olympus SZ60, Barcelona, Spain] to determine the mode of failure. Fractures were classified as adhesive, cohesive or mixed.

- Statistical analysis

First, a full-factorial regression model was used to assess the significance of the interaction between the three factors considered in this study [type of cement sealer, adhesive and root third].The Kolmogorov-Smirnov test was used to check the normality of data distribution. Because the results for each group did not follow a normal distribution, the variables were analysed using a non-parametric test. Pairwise comparisons of the microtensile bond strength means for the groups were analyzed using the Mann-Whitney U-test, and the Kruskal-Wallis test was used for global comparisons. The level of statistical significance was set at P<.05. The failure mode was analyzed using the Pearson χ² test.

## Results

The full-factorial regression model of the influence of the type of cement sealer [AH Plus™ and EndoREZ®], adhesive [no adhesive, Optibond Solo™ Plus and Clearfil Liner Bond 2V] and root third [coronal and middle] did not reveal any statistically significant interaction between the three factors [*p*=0.477]. Only the adhesive factor was significant in the regression model [*p*<0,001]; its effect on the microtensile bond strength is shown in  Table 2 along with the mean microtensile bond strength values.

**Table 2:**
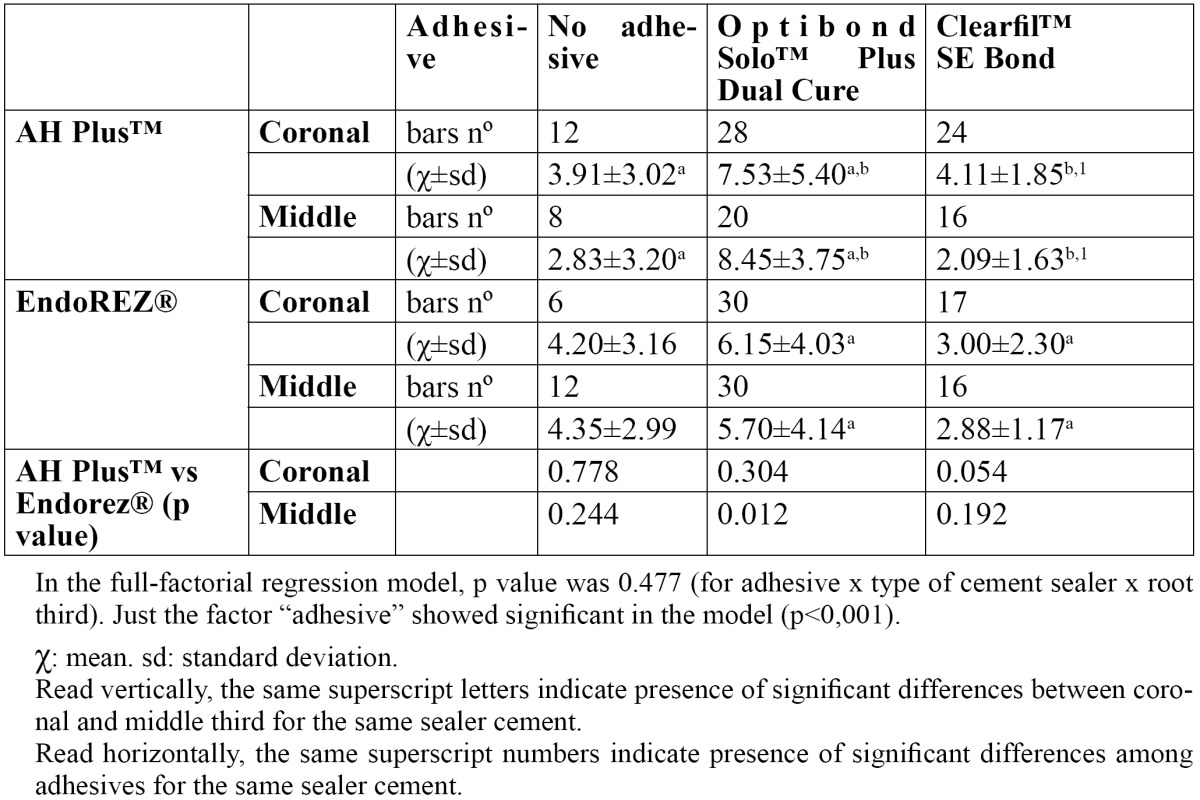
Comparison of the microtensile bond strength means among groups and regional influence for AH Plus versus EndoREZ.

The use of Optibond Solo™ Plus with AH Plus™ gave the highest mean microtensile bond strength values in both coronal [7.53±5.40] and middle thirds [8.45±3.75]. Similarly, Optibond Solo™ Plus attained the best results when used in conjunction with EndoREZ®, both in the coronal third [6.15±4.03] and in the middle third [5.70±4.14]. The lowest results were seen in the groups where no dentine adhesive was applied, and in those where the self-etching adhesive Clearfil Liner Bond 2V was used.

With AH Plus™, the results of the Optibond Solo™ Plus differed significantly from those of the group without adhesive [*p*=0.033], and the Clearfil Liner Bond 2V group [*p*=0.018], in the coronal third. Significant differences were also found for the middle third when comparing the group without adhesives and Optibond Solo™ Plus samples [*p*=0.004], and between Optibond Solo™ Plus and Clearfil Liner Bond 2V [*p*<0.001]. Comparison of the results between thirds showed that only Clearfil Liner Bond 2V gave statistically significant differences in the results obtained in the coronal third as opposed to the middle third [*p*=0.002].

In the groups where the sealer EndoREZ® was used, significant differences were found when Optibond Solo™ Plus was compared to Clearfil Liner Bond 2V, in both the coronal third [*p*=0.003], and the middle third [*p*=0.006]. Comparison between thirds gave no significant differences in any category of adhesives.

When the results obtained with AH Plus™ cement sealer were compared to EndoREZ®, no significant differences were found between coronal and middle thirds in any adhesive category, except when Optibond Solo™ Plus was used in the middle third [*p*=0.012].

The failure modes are shown in  Table 3. The most common failure mode was mixed in coronal dentin, with both AH Plus™ and EndoREZ®; however there were statistically significant differences between the modes of failure [*p*<0.001]. In middle third, all modes of failures were mixed [*p*=1.000].

**Table 3:**
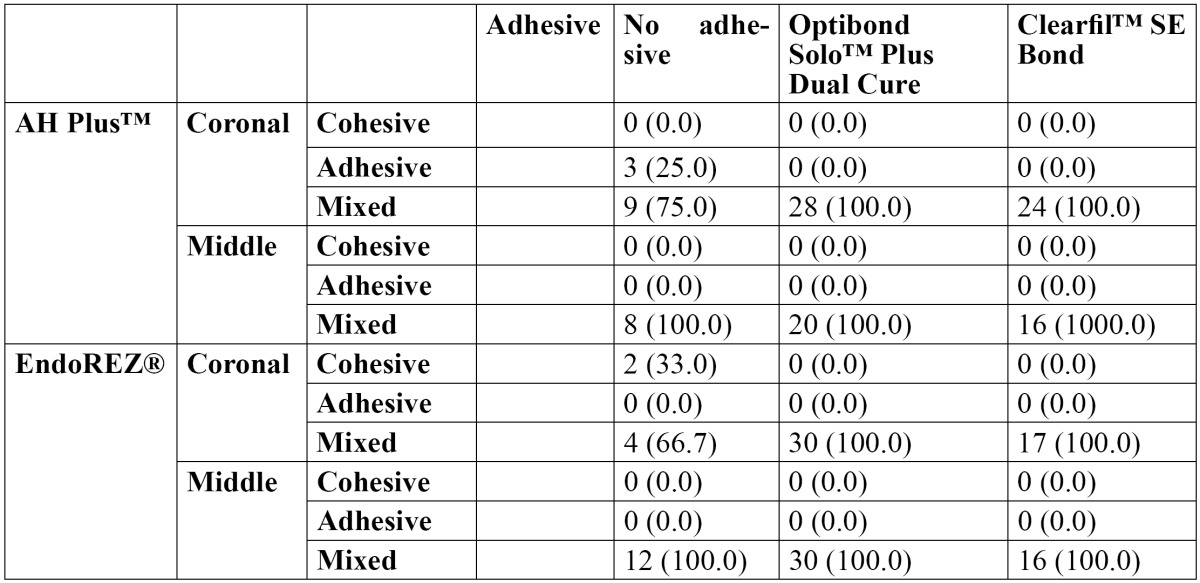
Mode of failure [n(%)].

## Discussion

Dentine bonding agents have been used before filling root canals in attempts to bond the root dentin to reduce apical and coronal leakage (7,8). In the present study, the microtensile bond strength to root dentin of AH Plus™ and EndoREZ® resin-based sealers, with or without use of Clearfil Liner Bond 2V and Optibond Solo™ Plus dentine adhesive systems, was tested on bovine teeth (16).

Although various methods have been used to measure the adhesión of endodontic sealers, including tensile strength tests, shear testing, microshear bond testing, microtensile bond strength and push-out tests, none of these have been generally accepted as a standard method for measurement. We chose µTBS technique for this study of adhesion because we can obtain many bars from a single tooth. In the Bolaños et al. study (17) eight to twelve 1 mm2 dentin/adhesive bars were obtained from each primary molar and only 4 (18) or 3 (19) slices trimmed into an hourglass shape were obtained from each human root specimen. We have obtained a much larger number of bars [even 60 bars when used Optibonb Solo Plus and EndoREZ®] because we used bovine roots split longitudinally in a mesio-distal direction. A major drawback of the µTBS technique is premature failure during specimen cutting (20). For that reason in the groups where we used etching adhesive we got many more bars than in the control group [18 with EndoREZ®]. µTBS [trimming and non-trimming] does not take account of the stress generated in 3-D cavities, leading to an overestimation of outcomes (21) so in our methodology we have considered the negative influence of the geometry of the cavity since we have reproduced the double adhesive interfaces because two halves of a single root were adhered to each other and we think that for this reason our results are lower.

It is know that the adhesión of methacrylate resin-based sealers to the root dentine was increased in the absence of smear layer (4,6) EDTA is usually recommended as the final rinse to remove the smear layer and this way reduce leakage and improve the seal of filled root canals (22). Eldeniz et al. (4) have informed that shear bond strength values of Diaket, AH Plus™ and EndoREZ® was increased in the absence of smear layer, where AH Plus™ showed the highest values of adhesion to dentine. These authors attribute their results to EndoREZ® co-hesive force is less than its adhesive strength. EndoREZ® is very effective in penetrating dentinal tubules and adapting closely to the canal walls the creation of long, unbonded resin tags alone (23). Exclusive adhesive failures were observed when EndoREZ® was used where the most of these tubules were empty because of pulling of the sealer resin tags out of the tubular orifices without the adhesive. The other hand, the adjunctive use of the self-etch adhesive with EndoREZ® resulted in higher tensile bond strengths, with fractures within hybrid layers and sealer tags (15). In our study, when dentine adhesive systems were not used, EndoREZ® showed slightly higher microtensile bond strength, although without significant differences with respect to AH Plus™. Therefore, the first null hypothesis can be accepted.

Effective bonding in the root canal environment remains a challenge and currently two adhesives strategies are commonly used [self-etch and total etch self-cure adhesives] (5,14). In this work we not found differences between the values obtained without adhesive or with the use of a self-etch adhesive, in return with the use of a total-etch adhesive the results were statistically higher. With the use of phosphoric acid the smear layer was completely dissolved and the exposed dentine was partially demineralized, so removing endodontic smear layer is an important step for effective increased bonding of methacrylate resin-based sealants to the radicular dentin (6,7).

In our study, the use of Optibond Solo™ Plus was seen to increase the µTBS of AH Plus™ and EndoREZ® to root dentine. The lowest values were obtained with Clearfil Liner bond 2V combined with the two resin-based sealers. Doyle et al. (14) and Gogos et al. (5) obtained the best results with the use of the self-etching system to bond EndoREZ® and AH-26 sealers respectively to root canal dentin. In contrast, we have found greater bond strength for an epoxy-resin sealer, AH Plus™, when the Optibond Solo™ Plus adhesive was used, as opposed to its use as the only filling material and lower bond strength was observed with the self-etching adhesive Clearfil Liner Bond 2V when compared with Optibond Solo™ Plus, for both cement sealers. This may be because the self-etching adhesive systems reduce the efficacy of the chemical polymerization of the resin-based sealers (24,25) facilitating the diffusion of moisture from the dentine and the creation of water bubbles in the den-tine-sealer interface, thus reducing the bond strength (26). Moreover, as Doyle et al. (14) claim, a mild self-etching adhesive [Clearfil Liner Bond 2V] placed on smear layer-covered dentine was not able to etch through thick dentine smear layer and bond to the underlying intact dentine. The primer ionization or solvent evaporation of the self-etching adhesives could affect the mechanical properties of the interface and play an important role in the final bonding to dentine (27).

Regarding the influence of the root area, Giannini et al. (15) studied the influence of tubular density on bond strength in different levels of the coronal dentin of two adhesive systems: Clearfil Liner Bond 2V and Prime & Bond 2.1. Their results showed that self-etching adhesive system was less influenced by the depth of dentine and tubular density than a total-etch adhesive. Foxton et al. (28) studied the adhesion to root coronal and apical dentine using one and two step adhesives. The strength of adhesion obtained was not dependent on the density of dentinal tubules. As Foxton et al. (28) we found no significant differences between the coronal and middle thirds in all groups when EndoREZ® sealer was used. However, AH Plus™ showed significant differences with Clearfil Liner Bond 2V, where the bond strength to dentine was higher in the coronal third that in the middle third.

The stronger bond strength obtained with the total etching system Optibond Solo™ Plus, in conjunction with both sealers, is most likely related with the formation of a wide resin-dentine interdiffusion zone, involved in the micromechanical interlocking of the adhesive materials and the etched root dentine (29). Resin-dentine hybridization at the surface of resin tags penetrating in many directions may contribute to a firmer bonding (1). In this context, total-etch adhesion has been shown to be stronger than self-etching adhesion, thus, the second null hypothesis had to be rejected.

The main objective of endodontic sealers is to get a total sealing but the polymerization shrinkage of methacrylate resin–based sealers inside long narrow canals, due to high cavity configuration factor (30) [C-factor], can disrupt the close initial contact between the sealer and the surrounding dentin and create a gaps where microor-ganisms can penetrate and grow as a biofilm (22). Although it has been reported low tensile bond strengths of methacrylate resin–based sealers to radicular dentin (20) the use of self-etching dentine bonding agents significantly reduced the apical microleakage of AH-26 sealer into root canal (8) and the total-etch technique may result in a higher bond strength in both pulp chamber and root canal dentin (18). We think that the use of a dual-cured total-etch adhesive could reduce the formation of gaps along of the sealer-dentin interface and create a hybrid layer with higher tensile bond strength to dentin. The stress can be released due that the filling materials behaving as elastomers and they can dissipate the stress polymerization instead of transmitting stresses to interface.

Under the conditions of this study, the use of a total-etch adhesive, Optibond Solo™ Plus, effectively increased the bonding strength of the sealers AH Plus™ and EndoREZ® to root dentin. The use of the self-etching Clearfil Liner Bond 2V adhesive did not improve the microtensile bond strength of both endodontic sealers. There was no significant differences in μTBS of a self-etching adhesive [Clearfil Liner Bond 2V] and no adhesive groups.
